# Autoimmune nodopathy with anti-contactin 1 antibody characterized by cerebellar dysarthria: a case report and literature review

**DOI:** 10.3389/fimmu.2024.1308068

**Published:** 2024-03-08

**Authors:** Jiajie Chen, Lingchun Liu, Hongyan Zhu, Jinming Han, Rong Li, Xiarong Gong, Hao Fu, Jingjing Long, Haixia Li, Qiang Meng

**Affiliations:** ^1^ Department of Neurology, The Affiliated Hospital of Kunming University of Science and Technology, The First People’s Hospital of Yunnan Province, Kunming, China; ^2^ Department of Clinical Laboratory, The Affiliated Hospital of Kunming University of Science and Technology, The First People’s Hospital of Yunnan Province, Kunming, China; ^3^ Department of Neurology, Xuanwu Hospital, Capital Medical University, Beijing, China; ^4^ Department of Magnetic Resonance Imaging, The Affiliated Hospital of Kunming University of Science and Technology, The First People’s Hospital of Yunnan Province, Kunming, China

**Keywords:** contactin-1, autoimmune nodopathy, cerebellar dysarthria, nerve conduction studies, magnetic resonance imaging

## Abstract

**Background:**

Autoimmune nodopathy (AN) has emerged as a novel diagnostic category that is pathologically different from classic chronic inflammatory demyelinating polyneuropathy. Clinical manifestations of AN include sensory or motor neuropathies, sensory ataxia, tremor, and cranial nerve involvement. AN with a serum-positive contactin-1 (CNTN1) antibody usually results in peripheral nerve demyelination. In this study, we reported a rare case of AN with CNTN1 antibodies characterized by the presence of CNTN1 antibodies in both serum and cerebrospinal fluid, which is associated with cerebellar dysarthria.

**Methods:**

A 25-year-old man was admitted to our hospital due to progressive dysarthria with limb tremors. The patient was initially diagnosed with peripheral neuropathy at a local hospital. Three years after onset, he was admitted to our hospital due to dysarthria, apparent limb tremor, and limb weakness. At that time, he was diagnosed with spinocerebellar ataxia. Eight years post-onset, during his second admission, his condition had notably deteriorated. His dysarthria had evolved to typical distinctive cerebellar characteristics, such as tremor, loud voice, stress, and interrupted articulation. Additionally, he experienced further progression in limb weakness and developed muscle atrophy in the distal limbs. Magnetic resonance imaging (MRI), nerve conduction studies (NCS), and autoimmune antibody tests were performed.

**Results:**

The results of the NCS suggested severe demyelination and even axonal damage to the peripheral nerves. MRI scans revealed diffuse thickening of bilateral cervical nerve roots, lumbosacral nerve roots, cauda equina nerve, and multiple intercostal nerve root sheath cysts. Furthermore, anti-CNTN1 antibody titers were 1:10 in the cerebrospinal fluid (CSF) and 1:100 in the serum. After one round of rituximab treatment, the patient showed significant improvement in limb weakness and dysarthria, and the CSF antibodies turned negative.

**Conclusion:**

Apart from peripheral neuropathies, cerebellar dysarthria (central nervous system involvement) should not be ignored in AN patients with CNTN1 antibodies.

## Introduction

Autoimmune nodopathy (AN) is a spectrum of motor-sensory peripheral neuropathies mediated by antibodies associated with adhesion molecules at the nodes of Ranvier and paranodes, including contactin-associated protein 1 (CASPR1), contactin-1 (CNTN1), neurofascin 155 (NF155) and neurofascin isoforms 140/186 (NF140/186) (1). AN has been considered a variant of chronic inflammatory demyelinating polyradiculopathy (CIDP). Emerging evidence supports the idea that AN has a specific clinical phenotype, with no significant macrophage-mediated demyelination and a poor therapeutic response to corticosteroid therapy and intravenous immunoglobulin (IVIg). In 2021, the European Academy of Neurology/Peripheral Nerve Society proposed the designation AN for these antibody-related disorders (2). Specifically, anti-CNTN1 AN was the first documented disease subtype, mainly in elderly individuals (3). Instead of the obvious inflammation or traditional macrophage-mediated demyelination resembling CIDP, anti-CNTN1 AN was found to cause detachment of terminal myelin from the axolemma in the paranode region, resulting in nerve conduction abnormalities similar to the diagnostic criteria for CIDP (4). Clinical manifestations of AN patients with CNTN1 antibodies mainly include symmetrical limb weakness, paresthesias, tremors, and sensory ataxia (5). Respiratory system and cranial nerve involvement (mainly facial paralysis, ophthalmoplegia, and diplopia) may also be noted (6, 7).

Reports of involvement of the central nervous system (CNS) in AN patients with anti-CNTN1 antibodies are rare. In our case, cerebellar dysarthria was noted in AN patients with anti-CNTN1 antibody, with positive CNTN1 antibody in the cerebrospinal fluid (CSF) providing strong evidence for possible CNS involvement. The patient, who was very young, also experienced distal limb weakness, sensory disturbances, postural dizziness, diminished tendon reflexes, irregular nystagmus, and ataxia. Nerve conduction studies (NCS) found chronic progressive peripheral nervous system damage involving myelin to axons. We followed the above with a review of the literature, which provided novel insights and values for early identification and in-depth investigation of AN with anti-CNTN1 antibodies in the future.

## Case presentation

A 20-year-old man was admitted to our hospital due to progressive dysarthria, slurred speech, and limb tremors for more than 8 years. He had no remarkable medical history. His first clinical symptoms were noted in April 2015, characterized by limb tremors, slurred speech, and mild limb weakness. He was initially evaluated for peripheral neuropathy at a local hospital and underwent a 5-day treatment of daily IVIg at a dose of 20g and methylprednisolone pulse therapy (initial dose 500mg). However, there was no significant improvement in clinical symptoms.

In 2018, the patient presented to our hospital with dysarthria, apparent limb tremor, and limb weakness. Dysarthria is specifically characterized by slurred speech, pauses in speech, and pronounced interruptions in pronunciation, and is accompanied by certain plosive sounds and abnormal intonation. The subject had no dysphagia, water aspiration during drinking, abnormal pharyngeal sensation, glossopharyngeal neuralgia, difficulty with tongue extension, or tinnitus. The NCS revealed that the conduction of multiple motor and sensory nerves was impaired (**Table 1**). At that time (before AN was widely recognized), a clinical diagnosis of CIDP was considered. However, the patient’s symptoms did not improve significantly following corticosteroid therapy and IVIg. Therefore, careful evaluation should be performed to rule out spinocerebellar ataxias (SCA) associated with peripheral neuropathy.

In the following five years, the patient developed worsening lower limb weakness, postural dizziness, muscle atrophy, and paresthesia in the lower limbs, along with distal numbness. Therefore, he was admitted to our hospital in March 2023. A neurological examination was performed on admission: the patient’s uvula and tongue extension were centered, the soft palate could be raised, the pharyngeal reflex was present, and there was no facial paralysis with normal hearing. Dysarthria was observed, and both eyes showed irregular nystagmus. His lower limb muscle strength was graded at 3/5. The tendon reflex of the upper limbs was weakened, while the knee reflex, distal pain, and vibratory sensation were decreased. Both the nose‐finger and heel‐shin tests were inaccurate. Bilateral Babinski and Kernig signs were negative.

**Figure 1:**
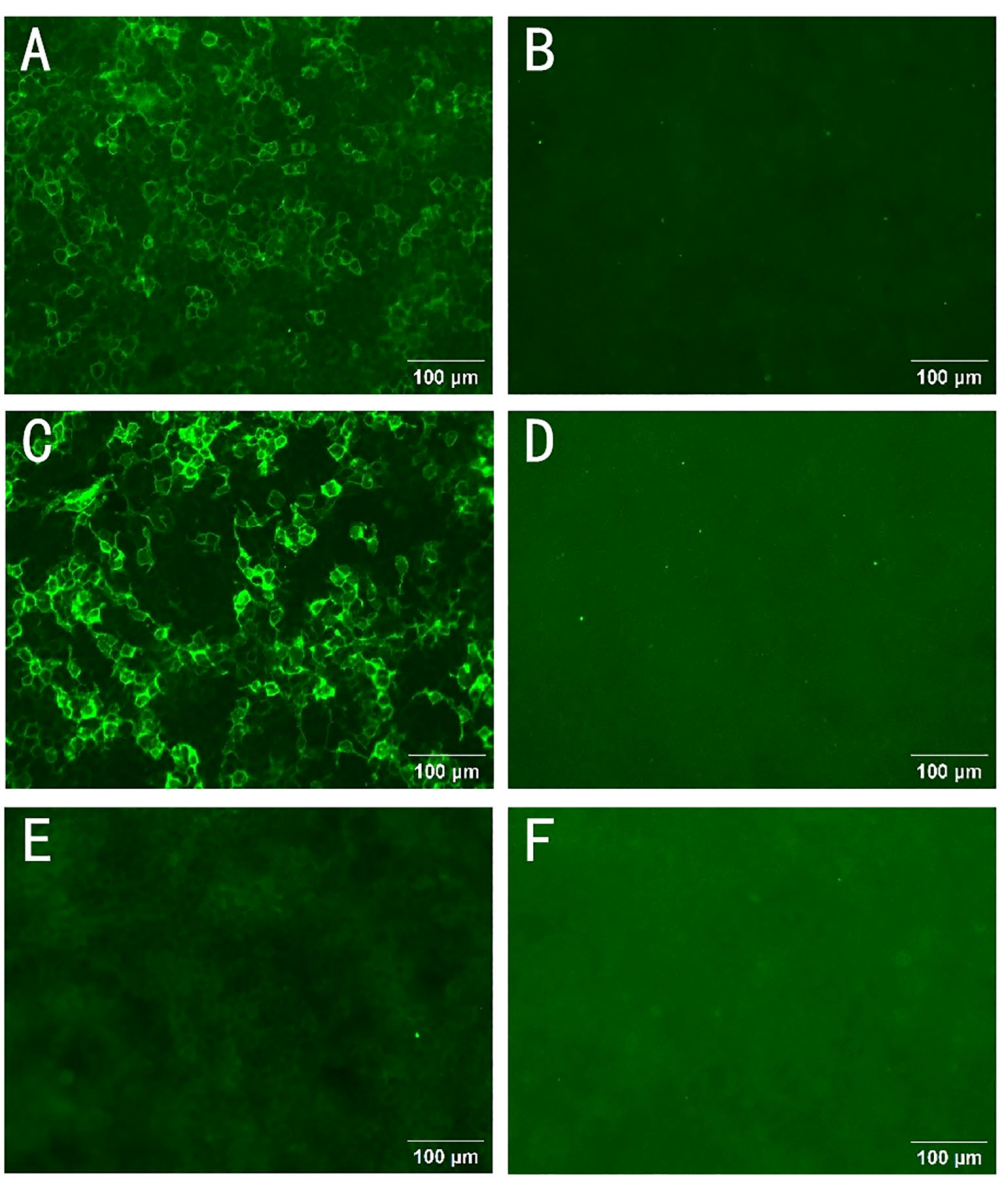
Autoantibodies to CNTN1 in serum and cerebrospinal fluid. The presence of anti-CNTN1 antibodies was confirmed using a cell-based assay (CBA) with CNTN1-transfected Human Embryonic Kidney (HEK) 293 cells. Cells overexpressing CNTN1 antigen on cell slides were able to bind to the patient’s anti-CNTN1 autoantibody IgG4. The antibody bound to the cells on the slides could be detected by a Fluorescein Isothiocyanate (FITC) labeled anti-human IgG4 secondary antibody. Finally, its reactivity was observed and analyzed by immunofluorescence microscopy. A positive CNTN1 antibody reaction was noted in the cerebrospinal fluid (CSF) **(A)**, the CSF control was negative **(B)**, the positive serum CNTN1 antibody was detected **(C)**, and the serum control was negative **(D)**. After one round of rituximab treatment, the patient’s CSF CNTN1 antibody tests were negative **(E)**. Negative control group **(F)**. Scale bar:100 μm.

**Figure 2:**
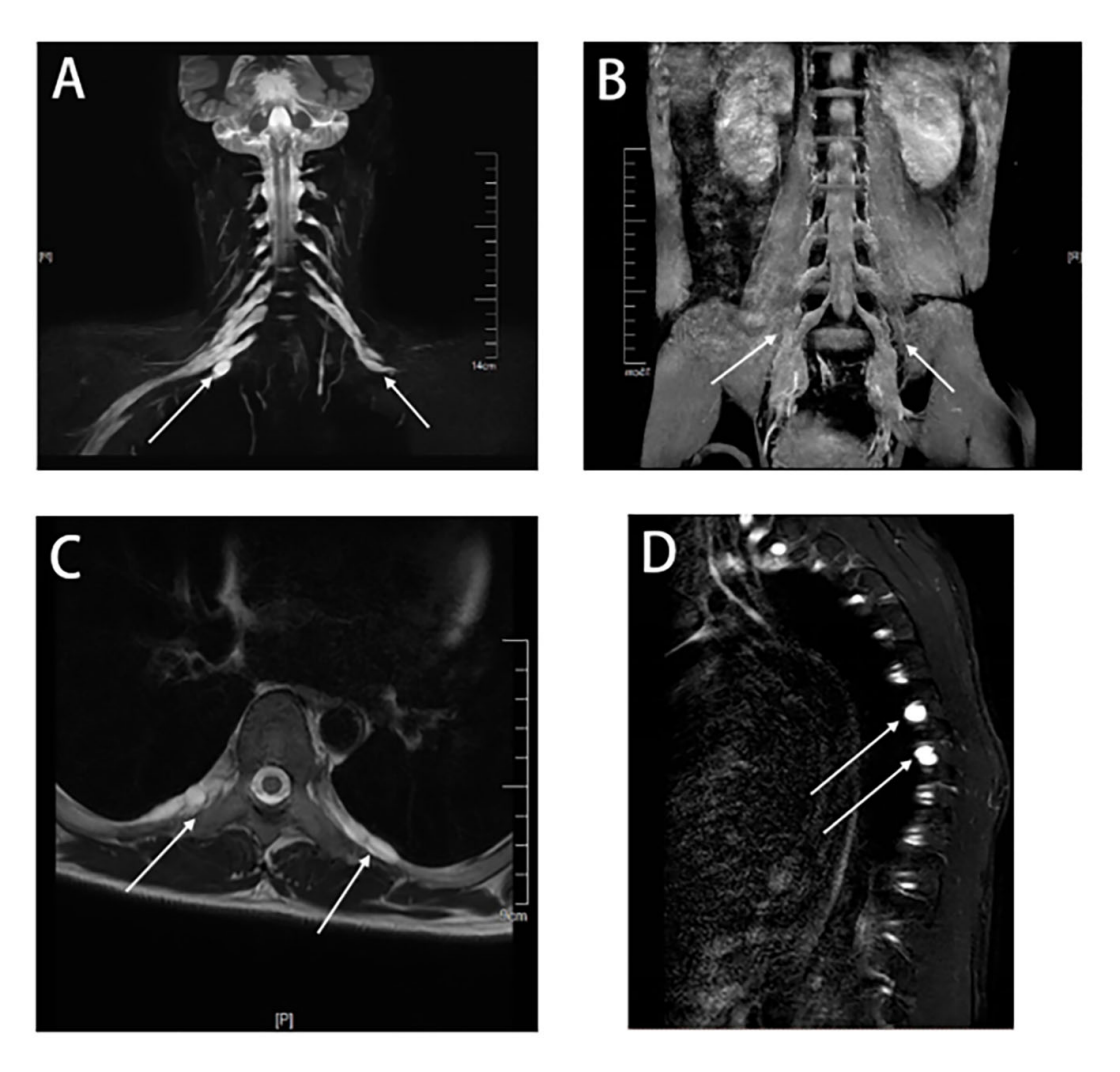
MRI of the cervical, thoracic, and lumbar spines of this patient. The principle of selective excitation technique (PROSET) of thin coronal MRI **(A)** showed diffuse thickening of nerve roots in the posterior segment of the cervical plexus, and three-dimensional short-time inversion recovery (3D-STIR) magnetic resonance imaging **(B)** showed diffuse thickening of nerve roots in the posterior segment of the lumbar plexus. A thoracic axial T2-weighted plain scan **(C)** showed nerve root sheath cysts in the intercostal nerves. Multiple nerve root sheath cysts were observed in T2-weighted fat-suppressed sequences **(D)**.

**Figure 3:**
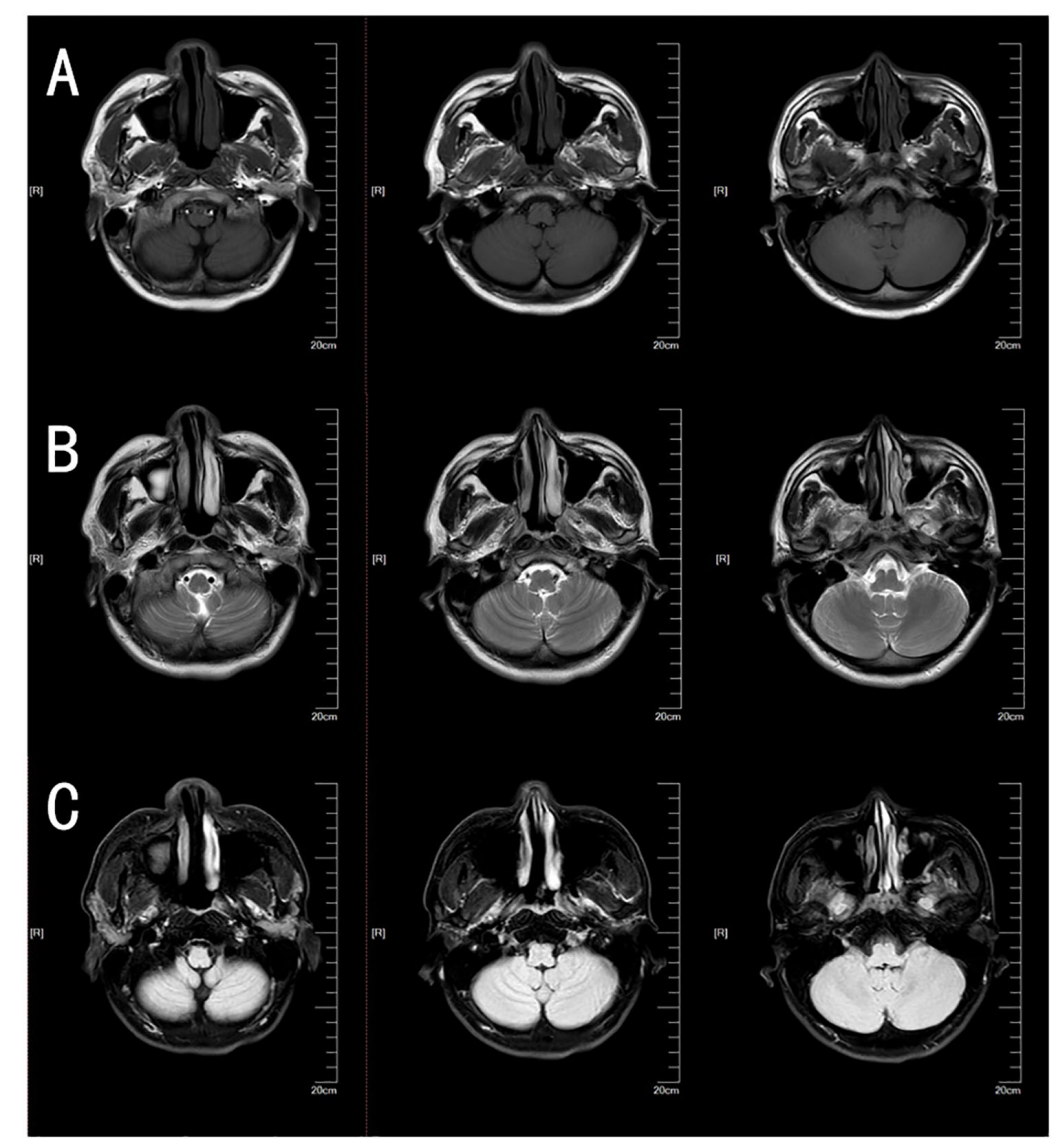
Brain MRI of this patient. The patient’s brain T1 plain scan sequence **(A)**, T2 plain scan sequence **(B)**, and fluid-attenuated inversion recovery (FLAIR) **(C)** did not reveal obvious cerebellar atrophy or other abnormal findings.

The NCS revealed the presence of motor and sensory nerve conduction abnormalities in multiple peripheral nerves. The Electromyogram (EMG) from five years earlier revealed denervation in the tibialis anterior muscle of the lower limb. However, after five years with no treatment, new findings of spontaneous potentials in the lower limbs and signs of denervation of the abductor digiti minimi in the upper limbs were identified in addition to the previously observed features. This indicated a wider extent and further progression of the denervation damage, suggesting that both the myelin sheath and the nerve axons were damaged. A lumbar puncture was performed and the CSF protein level was found to be 3009 mg/L (normal range: 150-450mg/L). The number of nucleated cells was 12*10^6^/L (normal range: 0-8*10^6^/L). CSF IgG: 247mg/L (normal range: 0-34mg/L); CSF IgA: 29mg/L (normal range: 0-5.0mg/L); CSF IgM: 16.6mg/L (normal range: 0-1.3mg/L). Anti-CNTN1 antibody titers were 1:10 in CSF and 1:100 in serum (**Figure 1**). Cervical Spine MRI revealed diffuse thickening of bilateral posterior cervical plexus segments, while thoracic MRI showed nerve root sheath cysts on the left anterior rib at thoracic disc plane 6-7 and bilateral anterior ribs at thoracic disc plane 7-8. Lumbar MRI showed diffuse thickening of the lumbosacral nerve roots (**Figure 2**). His cranial MRI revealed no abnormalities (**Figure 3**). In addition, antinuclear antibodies were positive with a titer of 1:200. Genetic screening for SCA was conducted, and the results were negative. In addition, the following urinalysis results were higher than normal values: urine protein: 1 + 0.3g/L, urine KAP light chain: 12.6mg/L (normal range 0-7.13mg/L), urinary LAM light chain: 6.2mg/L (normal value 0-3.94mg/L), 24-hour urine protein quantification: 5109mg/24h (normal range 0-300mg), urine total protein: 1890.05mg/L (normal range 0-150mg/L), urine immunoglobulin: 29.0mg/L (normal range 0-9.6mg/L), urine transferrin: 23.80mg/L (normal range 0-2.2mg/L), urine microalbumin: 523.0mg/L (normal range 0-30mg/L), suggesting that the patient had impaired renal function.

A diagnosis of anti-CNTN1 positive AN was made. Despite three rounds of plasma exchange being used, there was no significant improvement. As an alternative, we administered a single low dose of 500mg Rituximab. Eight months later, the patient returned to our hospital. It was observed that his tremor, gait stability, and walking speed had improved. Muscle strength in the distal extremities improved from grade 3 to 4. Residual mild dysarthria (characterized by mild syllable prolongation and uneven pitch intensity) was noted, while postural dizziness and nystagmus essentially recovered. Additionally, anti-CNTN1 antibodies in the CSF became negative. Repeat urinalysis showed that the urine protein had become negative (**Figure 1**). NCS suggested partial improvement in the conduction of the tibial and common peroneal nerves in the lower limbs compared to before. The EMG indicated a decrease in spontaneous muscle activity (**Table 1**). These findings suggested that the patient experienced a partial recovery of nerve function.

**Table 1:** Nerve conduction studies (NCS)/Electromyogram(EMG) results from a patient with anti-CNTN1 autoimmune nodopathy.

Nerve detection	Time point detection			Normal value
Ulnar nerve	Detection 3 years after onset April 2018 Left/Right	Detection 8 years after onset March 2023 Left/Right	Eight months after rituximab treatment November 2023 Left/Right	
Motor nerve conduction velocity (m/s) Upper elbow - Wrist	43.4/30.0	19.3/16.6	21.8/15.5	≥50
Compound muscle action potential amplitude (mV) Upper elbow - Wrist	7.3/8.4	2.2/2.2	3.0/2.1	≥7.0
Motor terminal latency (ms) Wrist - ADM	5.35/5.07	6.27/5.21	6.87/5.81	≤4.0
Sensory nerve action potential amplitude (μV) Upper elbow - Wrist	Not elicited	Not elicited	Not elicited	≥7.1
Sensory nerve conduction velocity (m/s) Upper elbow - Wrist	Not recordable	Not recordable	Not recordable	≥50
F-wave latency (ms) Wrist - ADM	62.9/65.5	85.3/96.6	80.3/88.5	≤30
F-wave occurrence rate (%) Wrist - ADM	90.0/90.0	90.0/90.0	85.0/80.0	≥75
Median nerve				
Motor nerve conduction velocity (m/s) Elbow - Wrist	28.8/30.8	15.7/15.5	17.5/16.1	≥50
Compound muscle action potential amplitude (mV) Wrist - APB	9.7/8.1	4.0/1.2	4.1/1.1	≥6.0
Motor nerve distal latency (mS) Wrist - APB	6.04/6.42	8.85/9.00	9.06/11.6	≤4.2
Sensory nerve action potential amplitude (μV) Index finger - Wrist	Not elicited	Not elicited	Not elicited	≥9.5
Sensory nerve conduction velocity (m/s) Index finger - Wrist	Not recordable	Not recordable	Not recordable	≥50
F-wave latency (ms) Wrist - APB	60.1/65.7	89.4/131.0	91.6/111	≤30
F wave occurrence rate (%) Wrist - APB	90.0/90.0	95.0/40.0	95.0/75.0	≥75
Tibial nerve				
Motor nerve conduction velocity (m/s) Popliteal fossa - Ankle	30.1/31.8	Not recordable/13.3	28.2/31.7	≥40
Compound muscle action potential amplitude (mV) Ankle - AH	1.10/1.09	Not elicited/1.04	1.0/1.83	≥7
Motor nerve distal latency (ms) Ankle - AH	9.77/9.55	Not elicited/11.5	11.9/9.3	≤5
Sensory nerve action potential amplitude (μV)	Not checked	Not checked	Not checked	≥0.4
Sensory nerve conduction velocity (m/s)	Not checked	Not checked	Not checked	≥35.1
F-wave latency (ms) Ankle - AH	Not elicited	Not elicited	131.0/94.6	≤51.0
F wave occurrence rate (%) Ankle - AH	Not elicited	Not elicited	50.0/75.0	≥75.0%
Common peroneal nerve				
Motor nerve conduction velocity (m/s) Capitulum fibulae - Ankle	33.9/35.2	Not elicited	32.8/31.9	≥45
Compound muscle action potential amplitude (mV) Ankle - EDB	0.57/0.24	Not elicited	0.44/0.33	≥3
Motor nerve distal latency (ms) Ankle - EDB	9.35/10.4	Not elicited	8.45/9.34	≤5
Sural nerve				
Sensory nerve conduction velocity (m/s) Middle leg - Lateral malleolus	Not elicited	Not elicited	Not elicited	≥50
Sensory nerve action potential amplitude (μV) Middle leg - Lateral malleolus	Not elicited	Not elicited	Not elicited	≥3.3
Electromyogram				
Left abductor digiti minimi				
Average amplitude (μV)	502	1843	Not checked	≤1000
Average time limit (ms)	10.2	13.0	Not checked	≤9.5
Resting potential	Not elicited	Not elicited	Not checked	–
Left tibialis anterior muscle				
Average amplitude (uV)	2408	1377	1118	≤1000
Average time limit (ms)	16.0	15.0	16.0	≤12.1
Resting potential	Not elicited	Lead out P++, F+	Lead out P+	–

μV, microvolt; mV, millivolt; m/s, meter per second; ms, millisecond; ADM, abductor digiti minimi; APB, abductor pollicis brevis; AH, abductor hallucis; EDB, extensor digitorum; Resting potential: P, positive shape potential; F, fasciculation potential. Compound muscle action potentials were measured from peak to peak.

## Discussion

A unified understanding of AN has emerged in the last three years. In 2021, the European Academy of Neurology/Peripheral Nerve Society issued guidelines that proposed four types of antibody-related disease subtypes including CNTN1, NF155, Caspr1, and NF140/186 (8). Previous studies suggested that anti-CNTN1 positive AN leads to peripheral nerve damage with subacute or acute CIDP-like manifestations, accompanied by sensory ataxia, tremor, or cranial nerve involvement (1, 9). In this study, we report a rare case that presented with typical cerebellar dysarthria at the onset of the disease, which has not been reported in the literature. Generally, dysarthria can be divided into atony, spasticity, hypokinesia, hyperactivity, ataxia, and mixed dysarthria (10). Among them, ataxic dysarthria can usually be observed in cerebellar diseases, manifesting as syllable explosions, impaired rhythms, and pauses after syllable. The expression of CNTN1 is not only limited to the paranode, but it can also be found in dorsal root ganglion neurons and cerebellar granule neurons. A previous study indicated that CNTN1 can be detected in the retina, spinal cord, cerebral cortex, hippocampus, and cerebellum (11, 12). There is no evidence of intrathecal synthesis of autoantibodies against accessory nodules (12), whereas increased levels of protein in the CSF are common in patients with anti-CNTN1 positive AN. This is most likely caused by the disruption of the blood−brain barrier and peripheral circulating proteins may be infiltrated into the CNS (12). In animal and basic experiments, the CNTN1 antibody was confirmed to bind to the hippocampal neurons and the cerebellum of rats (3, 13), which provides certain theoretical support for our case. We summarized the specific clinical characteristics of anti-CNTN1 antibody-positive patients from previous relevant literature (details are in **Table 2**). It is worth noting that our case initially presented with dysarthria and limb tremors, along with irregular nystagmus, postural dizziness, and nerve root sheath cysts. These manifestations are rarely observed in cases with positive anti-CNTN1 antibodies previously reported in the literature. Research has shown that long-term exposure to anti-CNTN1 autoantibodies results in reduced expression of CNTN1 in cerebellar granule neurons and has identified a cytotoxic effect on cerebellar neurons (12, 32). Regarding the mechanism of anti-CNTN1 affecting the cerebellum, studies have shown that CNTN1 controls synaptic interactions between cerebellar interneurons, and the presence of antibodies may lead to disruption of this process (33). CNTN1 can facilitate the activity of the non-receptor tyrosine kinase Fyn (which is predominantly expressed in the cortex, cerebellum, hippocampus, and other areas of the central nervous system) by binding to the PTPα (receptor protein tyrosine phosphatase) protein, thereby promoting the development, function, and synapse formation of the central nervous system (11, 34, 35). However, the presence of CNTN1 antibodies can disrupt this process. Furthermore, studies have shown that the PTPα protein can also increase the activity of the non-receptor tyrosine kinase Src (34, 36). The literature indicates that concurrent deficiency of Src and Fyn kinases can lead to damage in cerebellar Purkinje cells, resulting in cerebellar injury (37). Therefore, we hypothesized that CNTN1 antibodies might cause cerebellar damage by inhibiting the interaction between CNTN1 and PTPα protein, thereby suppressing the activities of Fyn and Src kinases.

**Table 2:** Detailed clinical features and imaging features of anti-CNTN1 antibody-positive cases.

Author/References	Time	Total Number	Age /sex	Clinical features									Elevated CSF protein	Serum Anti-CNTN1 antibody	CSF Anti-CNTN1 antibody	Imaging features	
				Ataxia (Number of cases)	Weakness of the limbs(Number of cases)	Paresthesia(Number of cases)	Muscle atrophy (Number of cases)	Difficulty swallowing(Number of cases)	Tremor (Number of cases)	Postural dizziness(Number of cases)	Nystagmus (Number of cases)	Cerebellar dysarthria(Number of cases)				Nerve root thickening on MRI	Nerve root cyst
Appeltshauser et al. (14)	2020	3	67(median)/F	2	3	3	–	–	–	–	–	–	3	3	–	–	–
Xu et al. (15)	2021	1	57/M	1	1	1	–	–	–	–	–	–	–	1	–	–	–
Hashimoto et al. (16)	2018	1	70/M	–	1	1	1	–	–	–	–	–	/	1	/	1	–
Hou et al. (17)	2022	2	52(median)/M	2	2	2	–	1	2	–	–	–	2	2	1	2	–
Doppler et al. (13)	2015	4	/	–	4	4	/	–	3	–	–	–	4	4	/	/	/
Tan et al. (18)	2022	1	37/M	1	1	1	–	–	–	–	–	–	1	1	–	/	/
Tang et al. (19)	2023	4	38(median)/ 2/2(F/M)	3	4	4	–	–	–	–	–	–	3	4	–	/	/
Li et al. (5)	2023	1	62/M	1	1	1	–	1	–	–	–	–	1	1	1	1	–
Fukushima et al. (20)	2022	1	77/M	–	–	1	–	–	–	–	–	–	1	1	–	/	–
Santoro et al. (21)	2022	1	73/F	1	1	–	–	–	–	–	–	–	/	1	–	/	/
Taieb et al. (22)	2019	1	75/M	1	1	1	–	–	–	–	–	–	1	1	–	–	–
Delmont et al. (23)	2020	10	63(median)/2/8 (F/M)	9	8	10	/	/	/	–	–	–	–	10	–	–	–
Carrera-García et al. (24)	2019	1	2/M	–	1	–	–	–	–	–	–	–	1	1	1	–	–
Lin et al (25)	2018	1	20/F	1	1	1	1	–	–	–	–	–	1	1	–	1	–
Dubey et al. (26)	2020	10	61(median)/5/5 (F/M)	9	8	9	/	–	1	–	–	–	7	10	/	/	/
Liberatore et al. (27)	2022	4	53(median) 1/3 (F/M)	3	4	4	/	–	–	–	–	–	4	4	/	/	/
Pascual-Goñi et al. (6)	2021	15	58(median)/5/10 (F/M)	12	15	8	/	6	10	–	–	–	15	15	–	/	/
Plaisier et al. (28)	2021	1	61/M	1	1	1	/	–	–	–	–	–	1	1	/	–	–
Fehmi et al. (29)	2023	15	59(median)/12/3(F/M)	14	14	8	/	/	14	/	/	/	11	15	–	/	/
Miura et al. (30)	2015	13	61(median)/ 3/10(F/M)	13	13	13	–	1	2	–	1	–	1	13	–	–	–
Cortese et al. (9)	2020	3	56(median)/M	3	2	3	2	–	1	–	–	–	–	3	–	–	–
Papantoniou et al. (31)	2023	2	60(median)/M	2	2	2	–	–	–	–	–	–	–	2	–	–	–
Dong et al. (7)	2022	1	62/M	–	1	1	–	–	–	–	1	–	–	1	1	–	–
Querol et al. (3)	2013	3	71(median)/2/1 (F/M)	1	3	2	/	–	–	–	–	–	3	3	–	/	/
Our case	2023	1	23/M	1	1	1	1	1	1	1	1	1	1	1	1	1	1

CNTN1, contactin-1; F, female subject; M, male subject; CSF, cerebrospinal fluid; MRI, magnetic resonance imaging; -, none;/, unknown.

Patients with anti-CNTN1 AN were prone to kidney involvement and nephrotic syndrome (22). In our case, the 24-hour urinary protein level was significantly elevated. Additional elevated urine-related protein indicators suggested renal glomerular damage. In support of this, CNTN1 can also be expressed in podocytes, with IgG4 and CNTN1 antigens coexisting in glomerular deposition (21, 22).

When the patient presented to our hospital for the first time, a diagnosis of SCA was considered based on the results of NCS and his clinical presentation, suggesting that we should pay attention to the differentiation of NCS between anti-CNTN1 positive AN and SCA. The NCS of SCA with peripheral neuropathy is non-specific and may manifest as sensory and axonal neuropathy, sensorimotor axonal neuropathy, demyelinating sensorimotor neuropathy, and sensorimotor polyneuropathy with axonal and demyelinating sensations (38). NCS features of anti-CNTN1 positive AN include prolonged distal motor latency, slow nerve conduction velocity, conduction block, prolonged or absent F wave latency, decreased compound muscle action potential amplitude, slow nerve conduction velocity, and decreased sensory nerve action potential amplitude (39). Notably, the 5-year interval of NCS results in this case suggested progressive motor and sensory nerve damage, possibly due to secondary axonal damage after severe myelin sheath injury. After retreatment, NCS showed partial improvement in some lower limb nerves, suggesting that the treatment may be effective.

On an MRI of the spinal nerve roots in AN, the enhancement or thickening of the lumbosacral nerve roots can usually be noted (1). Our case showed enhancement not only in the lumbar nerve root, but also in the cervical plexus. At the same time, the patient had multiple nerve root sheath cysts in intercostal nerve roots near the thoracic vertebrae, which had not been previously reported in the condition of anti-CNTN1 positive AN. Previously, the disease was reported to mainly occur in middle-aged and elderly people (1). However, our case is a patient who had clinical symptoms at the age of 17, suggesting that anti-CNTN1 positive AN may also occur in young people. Further investigation is warranted to explore the possible divergent pathogenesis between middle-aged and elderly individuals.

Previous studies have shown that the majority of anti-CNTN1 antibodies belong to the IgG4 subclass (1). Notably, animal studies conducted by Labasque and colleagues have demonstrated that patient-derived IgG4 antibodies disrupt paranodal junction formation in an *in vitro* myelination assay in the absence of complement or inflammatory cells, suggesting that Anti-CNTN1 IgG4 antibodies may themselves be pathogenic (40). Anti-CNTN1 antibodies are produced by activated B cells. Rituximab is a monoclonal antibody directed against B cells that express high levels of CD20. The study has shown that patients treated with rituximab have a significant decrease in the proportion of circulating CD20^+^B cells, accompanied by a reduction in specific IgG4 antibodies of CNTN1 (17), which may be an important reason for the improvement of patients treated with rituximab in our case. Other studies have shown that CNTN1 patients have IgG3 antibodies instead of IgG4, and that they respond well to treatment with IVIg (41, 42). This may be because IVIg regulates the immune response mainly by binding to a variety of Fc receptors and activating the complement pathway, whereas IgG4 neither binds to the Fc receptor nor activates the complement pathway (43), so the action of IVIg fails.

In conclusion, in addition to peripheral neuropathies, cerebellar dysarthria (central nervous system involvement) should not be ignored in AN patients with CNTN1 antibodies.

## Data availability statement

The original contributions presented in the study are included in the article/supplementary material. Further inquiries can be directed to the corresponding authors.

## Ethics statement

The studies involving humans were approved by Ethics Committee of the First People’s Hospital of Yunnan Province. The studies were conducted in accordance with the local legislation and institutional requirements. The participants provided their written informed consent to participate in this study. Written informed consent was obtained from the individual(s) for the publication of any potentially identifiable images or data included in this article.

## Author contributions

JC: Writing – original draft. LL: Data curation, Resources, Writing – review & editing. HZ: Writing – review & editing. JH: Resources, Supervision, Validation, Writing – review & editing. RL: Writing – review & editing. XG: Formal analysis, Writing – review & editing. HF: Writing – review & editing. JL: Writing – review & editing. HL: Writing – review & editing. QM: Writing – review & editing.
